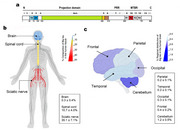# Distribution of big tau isoforms in the human central and peripheral nervous system

**DOI:** 10.1002/alz70855_105528

**Published:** 2025-12-24

**Authors:** Soumya Mukherjee, Rama Koppisetti, Nicolas R. Barthélemy, Kanta Horie, Cindy V. Ly, Justin Melendez, Timothy M. Miller, Chihiro Sato, Nupur Ghoshal, Celeste M. Karch, Randall J. Bateman

**Affiliations:** ^1^ Washington University School of Medicine, St. Louis, MO, USA; ^2^ Tracy Family SILQ Center, St Louis, MO, USA; ^3^ The Tracy Family SILQ Center, St. Louis, MO, USA; ^4^ Department of Neurology, Washington University in St. Louis School of Medicine, St. Louis, MO, USA; ^5^ Washington University in St. Louis School of Medicine, St. Louis, MO, USA; ^6^ Washington University at Saint Louis, Saint Louis, MO, USA; ^7^ Tracy Family SILQ Center, St. Louis, MO, USA; ^8^ Washington University School of Medicine, St Louis, MO, USA; ^9^ Department of Psychiatry, Washington University in St. Louis School of Medicine, St. Louis, MO, USA

## Abstract

**Background:**

Microtubule associated protein tau (MAPT) plays a crucial role in multiple neurodegenerative diseases such as Alzheimer disease (AD) and frontotemporal dementia (FTLD). While six isoforms are known in the central nervous system (CNS), the peripheral nervous system (PNS) expresses longer ‘big tau’ isoforms. Despite the knowledge of the tau mRNA transcript, ‘big tau’ protein distribution across the human nervous system and pathophysiological role remains largely unknown.

**Methods:**

We developed an immunoprecipitation coupled with mass spectrometry (IP/MS)‐based assay using antibodies binding to distinct regions of human tau (anti‐tau monoclonal antibodies HJ8.5, HJ8.7 and Tau1). We analysed postmortem brains (frontal, temporal, parietal, occipital and cerebellum) from AD (*n* = 17), disease control (*n* = 10) and Amyotrophic lateral sclerosis (ALS) (*n* =  8); postmortem spinal cord tissue (lumbar and cervical) from disease controls (*n* = 4) and ALS (*n* = 4) and sciatic nerves from ALS (*n* = 5) and disease control (*n* = 1).

**Results:**

Using tandem mass spectrometry, we discovered a novel human big tau isoform resulting from the inclusion of exon 4a‐long (exon 4a‐L), encoding 355 amino acids into the tau protein sequence. We demonstrated this exon 4a‐L to be the sole exon 4a variant expressed in humans, unlike the previously reported exon 4a. We found big tau also has distinct isoforms due to alternative splicing of the tau gene. This expands the number of human tau isoforms from six to twelve, significantly increasing the complexity of tau protein biology and greater diversity of its functional roles across human nervous system. Finally, we observed a central‐to‐peripheral gradient of big tau expression, lowest in the cortical brain regions (<0.5 %), followed by the cerebellum (1 %), then spinal cord (10 %), with the highest level in the human sciatic nerve (35 %).

**Conclusions:**

Our study provides the first protein‐level evidence for expanded repertoire of tau isoforms in the human nervous system. Interestingly, brain regions overlapping with relatively lower big tau are more susceptible to neurofibrillary tangle formation, suggesting divergent roles for different tau isoforms in disease. Our findings provide new insights into the basic biology of big tau in humans, crucial for understanding its pathophysiological functions.